# Construction of a high-density genetic map: genotyping by sequencing (GBS) to map purple seed coat color (*Psc*) in hulless barley

**DOI:** 10.1186/s41065-018-0072-6

**Published:** 2018-11-17

**Authors:** Xiaohua Yao, Kunlun Wu, Youhua Yao, Yixiong Bai, Jingxiu Ye, Dezhao Chi

**Affiliations:** 1 State Key Laboratory of Plateau Ecology and Agriculture, Qinghai University, Xining, 810016 China; 2 Academy of Agricultural and Forestry Sciences, Qinghai University, Xining, 810016 China; 3 Qinghai Key Laboratory of Hulless Barley Genetics and Breeding, Xining, 810016 China; 4 Qinghai Subcenter of National Hulless Barley Improvement, Xining, 810016 China

**Keywords:** Hulless barley·genotyping-by-sequencing (GBS), Purple seed coat color (*Psc*), Linkage analysis

## Abstract

**Background:**

Colored hulless barley are more suitable in food processing compared to normal (yellow) varieties because it is rich in bioactive compounds and produces higher extraction pearling fractions. Therefore, seed coat color is an important agronomic trait for the breeding and study of hulless barley.

**Results:**

Genotyping-by-sequencing single-nucleotide polymorphism (GBS-SNP) analysis of a doubled haploid (DH) mapping population (Nierumuzha × Kunlun10) was conducted to map the purple seed coat color genes (*Psc*). A high-density genetic map of hulless barley was constructed, which contains 3662 efficient SNP markers with 1129 bin markers. Seven linkage groups were resolved, which had a total length of 645.56 cM. Chromosome length ranged from 60.21 cM to 127.21 cM, with average marker density of 0.57 cM. A total of five loci accounting for 3.79% to 23.86% of the observed phenotypic variation for *Psc* were detected using this high-density map. Five structural candidate genes (*F3’M*, *HID*, *UF3GT*, *UFGT* and *5MAT*) and one regulatory factor (*Ant1*) related to flavonoid or anthocyanin biosynthesis were identified..

**Conclusions:**

Five structural candidate genes and one regulatory factor related to flavonoid or anthocyanin biosynthesis have been identified using a high-density genetic map of hulless barley. This study lays the foundation for map-based cloning of *Psc* but provides a valuable tool for studying marker-trait associations and its application to marker-assisted breeding of hulless barley.

**Electronic supplementary material:**

The online version of this article (10.1186/s41065-018-0072-6) contains supplementary material, which is available to authorized users.

## Background

Hulless barley (*Hordeum vulgare* L. *var*. *nudum* Hook. f.) is a self-reproducing annual species that produces naked grains. Hulless barley is widely grown on the Qinghai-Tibet Plateau. It has served as a staple food for the Tibetan people since the fifth century CE [1]. The seed coat color is an important agronomic trait in crops due to its association with unique biological activity and function in healthcare [2]. Upon maturity, barley grains may display different pigmentations. Barley has a variety of seed coat colors, including yellow, blue, purple, and black [3]. The use of colored grains in cereal-based functional foods has been considered based on their high levels of natural antioxidants such as phenolic compounds, anthocyanins and essential amino acids [4]. Colored barley varieties have been found to be more suitable for health than the standard (yellow) variety because these produce pearling fractions that are rich in bioactive compounds [5]. The development of barley flour, which itself has antioxidant properties, is used in various mainstream foods such as breads, muffins, noodles, and pasta [6]. The breeding of colored barley varieties has become an increasingly pertinent issue in agriculture in recent years.

Seed coat color is thought to be associated with the synthesis of anthocyanins, which are flavonoids present in plants [7]. The yellow color of barley is attributed to proanthocyanidins synthesized in the seed coat (testa layer) [8]. The purple color is associated with anthocyanins synthesized in the pericarp and glumes [9]. In barley, a number of mutants that lack anthocyanins or proanthocyanidins (designated *ant* mutants) have been documented. The *Ant* loci, known as anthocyanin or proanthocyanidin synthesis genes, are classified as *Ant1* to *Ant30* [10]. Flavonoid biosynthesis and the associated metabolic pathways have been studied in barley [11]. Also some candidate genes have been identified. *Ant1* (*HvC1*), which is located on chromosome 7H, reduces stem anthocyanin content [12]. *Ant2* (*HvbHLH1*), which is located on chromosome 2HL, regulates anthocyanin pigmentation in the auricles, awns, and lemmata. It is not involved, however, in grain proanthocyanidin pigmentation in grains [13]. *Ant17* (flavanone 3-hydroxylase; *F3H*), which is located on chromosome 2HL, has been observed in pigmented tissues. It is not found in nonpigmented roots and stems [14]. *Ant18* (dihydroflavonol 4-reductase; *DFR*), which is located on chromosome 3HL, is involved in both proanthocyanidin and anthocyanin synthesis [14, 15]. *Ant28* (*Hvmyb10*), which is located on chromosome 3HL, specifically regulates proanthocyanidin synthesis for grain color and dormancy [10]. Although these genes that are involved in the biosynthesis of anthocyanins in barley have been identified, their association with seed color in hulless barley remains unclear.

Major obstacles to traditional methods for marker development include low efficiency and high cost of generating high-density genetic linkage maps [16]. High-throughput sequencing technology has enabled the determination of hulless barley genome sequences [17]. Therefore, accelerating the process and efficiency of molecular marker-assisted breeding in this plant species is important. A high-density genetic map is a valuable tool in genomic and genetic applications and especially in fine mapping [18, 19]. Genotyping-by-sequencing (GBS), a simple and relatively inexpensive procedure, has reduced the complexity of mapping because it is particularly suitable for a large number of samples in genetic map construction [20–22]. Recent advances in GBS technology have allowed the identification of numerous genetic molecular markers at a reasonable cost. This has promoted the development of several high-throughput single-nucleotide polymorphism (SNP) genotyping methods [23].

The present study generated a large double haploid (DH) population from a cross between the Nierumuzha and Kunlun10. The seed coat of Kunlun10 is yellow and that of Nierumuzha is deep purple. Dense marker data was obtained for 298 DH individuals using the GBS technology. We identified 3662 efficient SNP markers, which were ultimately refined into 1129 bin markers after screening. The seed coat color of individual plants was determined. This information was used to map the *Psc* of hulless barley. The candidate genes and significant loci detected in this study suggested that our approach is cost-effective for fine mapping and can identify rapidly other key phenotypic genes in hulless barley.

## Materials and methods

### Plant materials and phenotyping

**Fig. 1:**
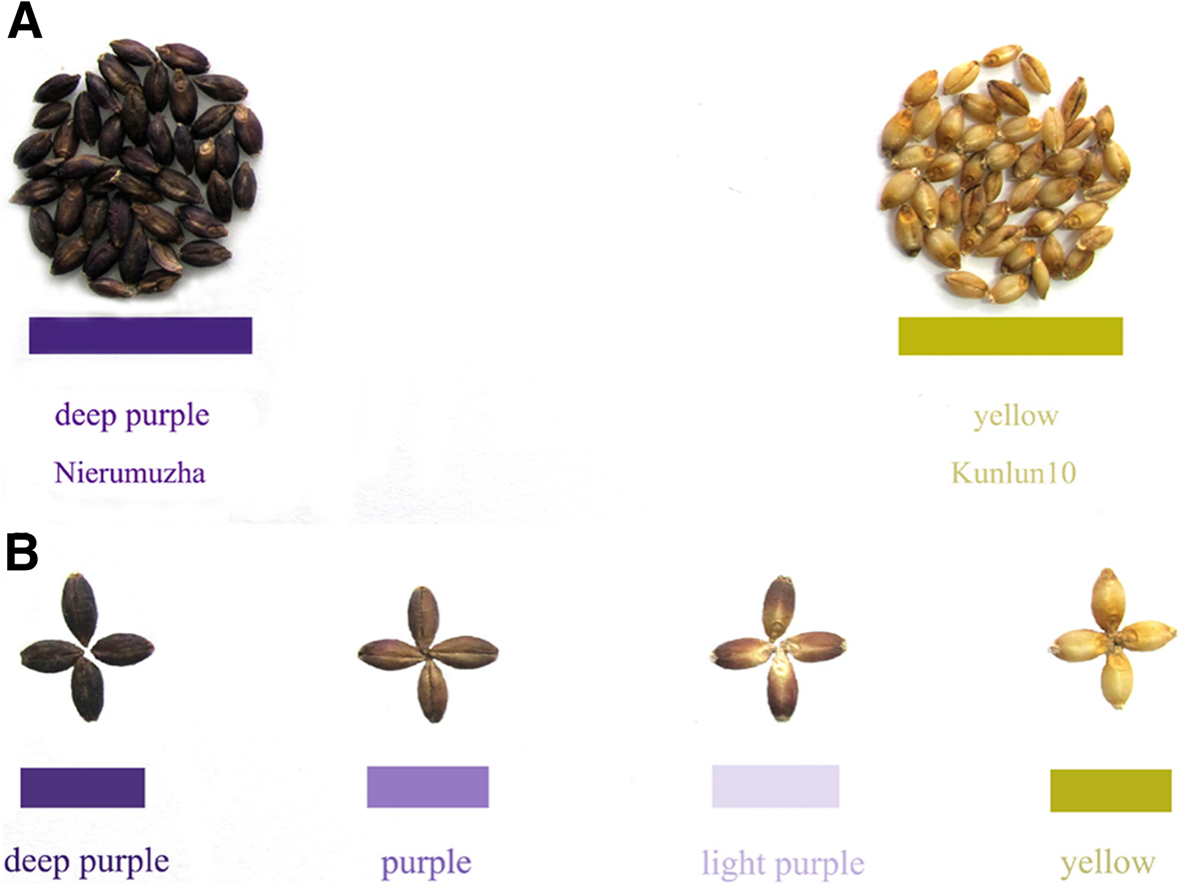
The phenotype pictures of female, male and four types of seed coat color. **a** The seed coat color of Nierumuzha and Kunlun10; **b** The phenotype of deep purple, purple, light purple, and yellow color seeds

A DH mapping population that consisted of 298 individuals was drawn from hulless barley Kunlun10 (yellow seed coat color) as males and Nierumuzha individuals (deep purple seed coat color) were drawn as females (Fig. 1a). Seed color was assessed using the Wanseen seed test system SC-G (Wseen, Hangzhou, China) combined with visual inspection. Mean trait values were averaged from up to three repeats from each genotype in the DH population. Linkage analysis of the seed color was derived from average color estimates.

### DNA extraction

Young leaves were collected from seven-week-old plants of parents and progeny after germination in April 2016. The leaves were immediately placed in liquid nitrogen immediately and then stored at − 80 °C. A plant genomic DNA kit (TIANGEN, Beijing, China) was used for genomic DNA isolation in accordance with the manufacturer’s protocol. DNA concentration and purity were assessed using a Nano Photometer® spectrophotometer (Implen, CA, US). DNA contamination and degradation were assessed on 1.0% agarose gels.

### Library construction

A genotyping-by-sequencing (GBS) (Novogene, Beijing, China) was used in this study to develop the SNP markers. A GBS pre-design experiment was performed. The GBS library was constructed using a double digest of genomic DNA with a combination of *HaeIII, MseI*, and *EcoRI* enzymes. This was subsequent to adapters with barcodes, after which each sample was amplified in multiplex with selected fragments for library construction. A tight length range was selected (about 50 bp) to maintain a uniform sequence depth among different fragments.

### Illumina sequencing

First, standard analysis of the raw data was conducted. The Illumina HiSeq™ sequencing platform (Illumina, San Diego, CA, USA) was employed for double-stranded (paired-end) 150-bp sequencing. Next, we conducted advanced analyses based on the assessment of the original data and DNA library assembly followed by HiSeq sequencing with removal of reads with low-quality base calls or uncalled bases. Second, we determined the number of reads digested by *Mse*I at both ends of each screened read in the progeny subjected to GBS-Seq analysis. We then discarded reads that did not contain these restriction sites. The specific reads were recorded as well as the ratio of the total number of reads to the number of enzyme captured reads. Finally, using an Illumina high-throughput sequencing platform, pair-end sequencing was performed on the selected tags, followed by SNP genotyping and evaluation followed.

### Reference genome mapping

For GBS, each sample was sequenced and then compared to the reference genome. Paired-end (PE) reads of the clean data from both the parent and offspring were compared to the reference genome by Burrows-Wheeler Aligner (BWA) software. SAM tools software [24] was used to create SAM/BAM format files, which were used to detect mutations, and coverage was determined using Perl scripts. A linkage map was constructed that was based on the hulless barley genome database (reference genome, ftp://ftp.ensemblgenomes.org/pub/release-29/plants/fasta/hordeum_vulgare/dna/).

### Sequence data analysis and SNP identification

The barcodes were used to sort the sequences of each sample. To ensure that the reads were without artificial bias and were reliable (low-quality paired reads, which are mainly caused by base-calling duplicates and adapter contamination), raw data (raw reads) in FASTQ format were first processed in the subsequent analyses in a series of quality control (QC) procedures using in-house C scripts. The QC standards were as follows: (1) removal of reads with > 50% bases having Phred quality < 5; (2) removal of reads with ≥10% unidentified nucleotides (N); (3) removal of reads containing the enzyme *HaeIII* and *EcoRI* enzyme restriction sites; and (4) Removal of reads with > 10 nt aligned to the adapter, allowing ≤10% mismatches.

SNP calling in parents and progeny was performed using the SAMtools software [25]. The SNPs and types of transversions or transitions were counted. A Perl script was then employed to filter the SNPs that had more than two genotypes. The parental polymorphic markers were arranged into eight segregation patterns (hk × hk, aa×bb, nn × np, cc × ab, ab×cc, lm × ll, ab×cd and ef × eg) according to the cross pollination (CP) model employed by JoinMap 4.0 software [26]. Segregation patterns were selected for the genetic mapping of offspring.

### Linkage map construction and linkage analysis

Prior to map construction, the markers with integrity > 75%, segregation distortion (*P* < 0.001), or that were in possession of abnormal bases were filtered. The segregation pattern aa × bb was used for map construction using JoinMap 4.0. To calculate the marker distances, a regression algorithm, three times circulation sequence, and Kosambi [27] mapping function were used. LOD values were within the range of 2.0–10.0. The integrated map was computed for the male and female parents using the combined group for map integration function in the MergeMap software. A Perl script SVG was used to visualize the exported maps. Heat maps were constructed to evaluate those maps. Linkage analysis was conducted using an LOD threshold of 1000 permutations and a *P* ≤ 0.05. A LOD score of 3.2 was set as the minimum for 1000 permutations to declare that any particular genomic region contained a locus. All candidate genes were categorized according to gene annotations by Swiss-Prot, TrEMBL, the Kyoto Encyclopedia of Genes and Genomes (KEGG) analysis.

## Results

### Resequencing of parental lines and GBS of the DH lines

**Fig. 2:**
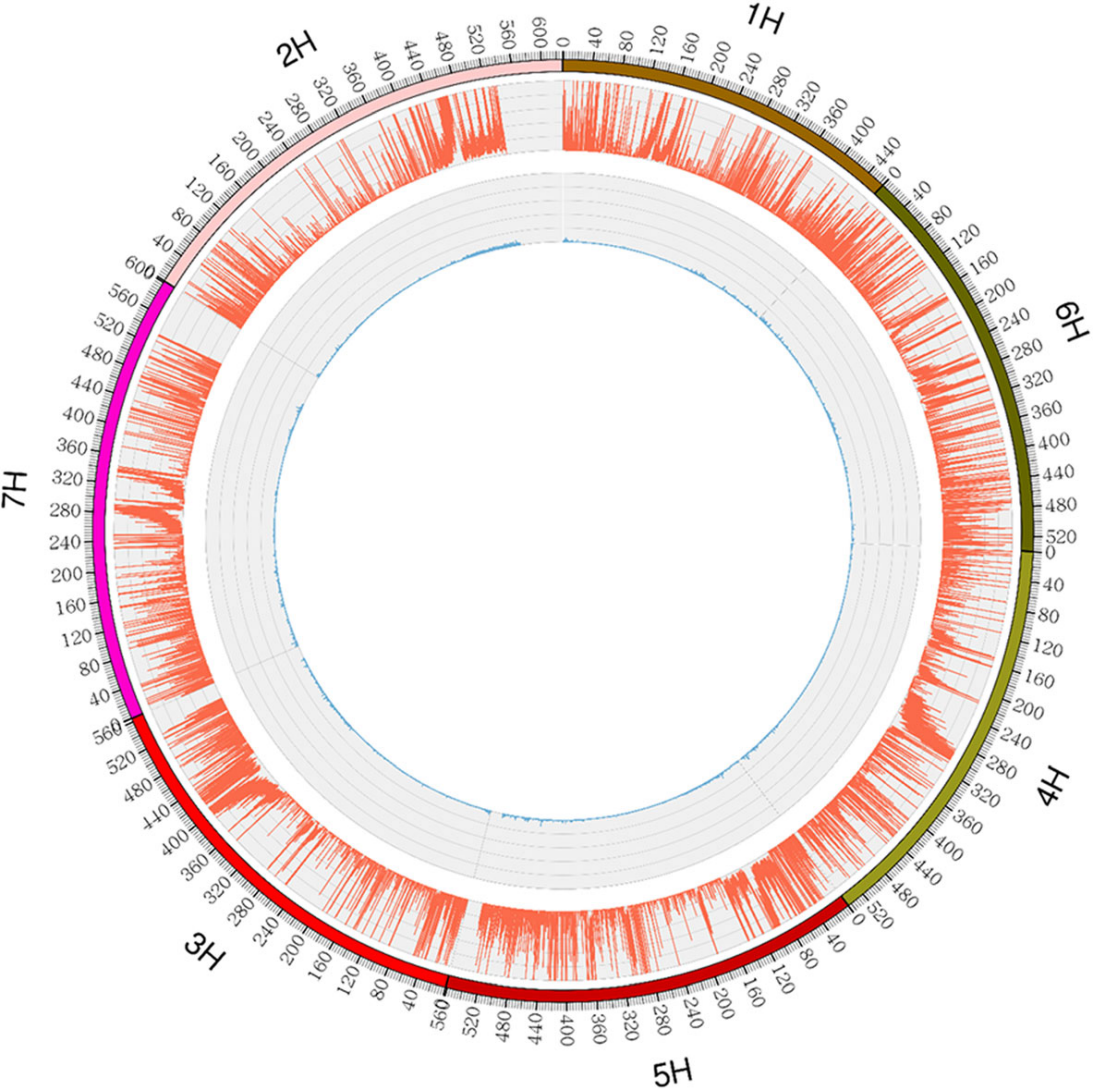
Distribution of SNPs and the aa × bb genotype throughout the Nierumuzha and Kunlun10 genome-wide. The outermost box that includes a scale represents the seven chromosomes of hulless barley. The orange histogram represents the density of SNPs that are polymorphic between Kunlun10 and Nierumuzha. The green histogram represents the density of aa × bb genotype SNPs that are polymorphic between Kunlun10 and Nierumuzha

The Nierumuzha and Kunlun10 parents were sequenced at effective sequencing depths of about 24.55-fold and 24.19-fold, respectively. 13,682,278 reads of Nierumuzha and 13,395,851 reads of Kunlun10 were mapped to the barley genome. The mapping rates were 96.81% and 96.72%, respectively (Table 1). Finally, 224,008 polymorphic loci were identified. The marker genotype data are summarized in Table 2. Only the genotype aa × bb, consisting of 20,615 markers between Nierumuzha and Kunlun10, was used in the subsequent for further analysis (Fig. 2).

**Table 1:** Sequence depth and coverage statistics

Sample	Clean reads^a^	Mapping reads^b^	Mapping rate ^c^	Average depth^d^	Coverage1×^e^	Coverage4×^f^
Kunlun10	13,850,762	13,395,851	96.72	24.19	8.33	4.33
Nierumuzha	14,133,242	13,682,278	96.81	24.55	8.39	4.37

^a^Number of reads used for the alignment

^b^Number of clean reads that mapped to the reference genome

^c^The percentage of reads that mapped to the genome

^d^Average sequencing depth

^e^Percentage of the reference genome with at least 19 coverage

^f^Percentage of the reference genome with at least 49 coverage

**Table 2:** Marker types

Marker type^a^	Numbers^b^
hk × hk	141,005
aa×bb	20,615
nn × np	30,054
cc × ab	7
ab×cc	6
lm × ll	30,310
ab×cd	1
ef × eg	2010
Total markers:	224,008

^a^Parental genotypes (i.e., ab × cc, where ab and cc genotypes represent the male and female parents)

^b^Total number of markers of each type

The DH lines were then genotyped using the GBS technology. The average number of raw individual reads of the 298 hulless barley lines was about 1.6 Gb. This yielded 490.07 Gb of high-quality sequence reads (Q20 ≥ 95%, Q30% ≥ 88%) and a normal GC content among the reads. The average *Mse*I enzyme capture rate was 93.14% across the GBS of the 298 DH lines, indicating that enzyme digestion was high quality. The average coverage of the 298 offspring was 96.93% of the whole genome, with 21.11 read depth at 8.38% (coverage at least 1×) and 3.76% (coverage at least 4×) of sites. The screened genotypes contained all of the markers in more than 75% of individual lines. Therefore, at least 227 of the 298 progeny lines contained all the markers.

### Genotyping of the progeny and selection of genetic markers

The low-coverage sequences of the DH lines (coverage under 75%) were filtered out. This left 7028 markers out of the original 20,615. Markers with significant distortion (*P* < 0.001) were filtered, and a total of 1549 markers were retained in total for identification of bin markers.

### Genetic linkage map with bin markers

**Fig. 3:**
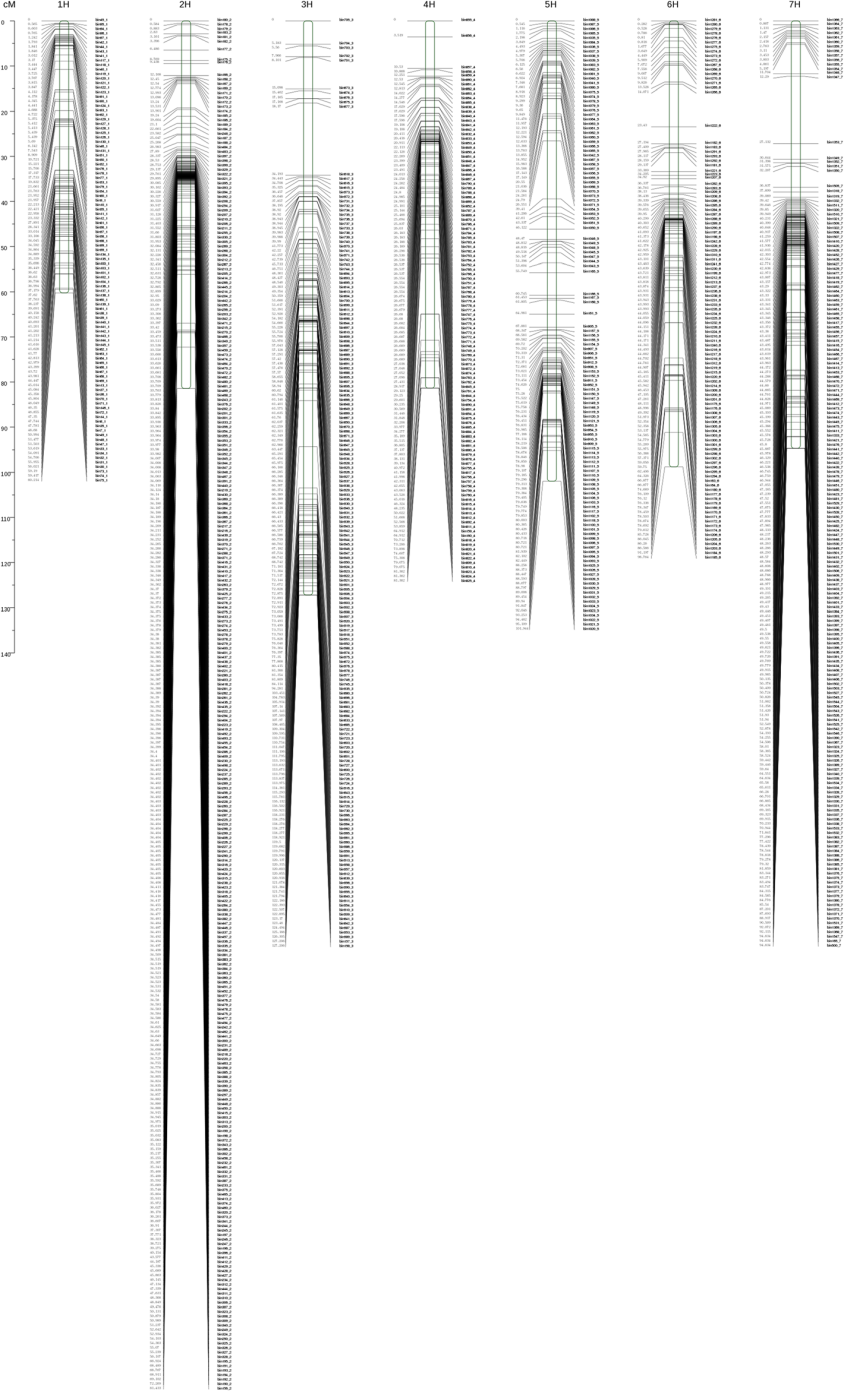
Distribution map of the linkage groups. Note, the x-axis is the chromosome number, the y-axis is the genetic distance (in cM), and the black line is the bin marker

Unlinked markers were filtered out, and 3662 SNPs (1129 bin markers) were mapped to seven linkage maps using Joinmap 4.0. A high-density genetic map was constructed after these 1129 bin markers were mapped onto the seven chromosomes of hulless barley. The genetic maps were 645.56 cM in length with an average distance of 0.57 cM between the markers. 3H was the largest group among the seven linkage groups, consisting of 182 markers, and a genetic length of 127.21 cM. 1H was the smallest group, with 103 markers, and a genetic length of 60.21 cM (Table 3, Fig. 3). A total of 1124 gaps were detected between markers. Among these, 11,102 gaps (98.04%) were < 5 cM, 15 gaps were between 5 and 10 cM in size, and only seven gaps were between 10 and 20 cM in size. However, there were no gaps over 20 cM in size were observed in any of the chromosomes (Table 4).

**Table 3:** Genetic linkage group statistics

Chr^a^	PD(bp)^b^	Bin markers^c^	SNP^d^	GD(cM)^e^	Average distance(cM)^f^	Max. gap (cM)^g^
1H	464,124,043	103	230	60.21	0.58	4.67
2H	628,342,783	301	1340	81.43	0.27	10.82
3H	564,427,874	182	406	127.21	0.70	16.02
4H	544,168,226	117	312	81.36	0.70	11.05
5H	561,411,686	127	240	101.94	0.80	12.86
6H	538,755,036	111	541	98.76	0.89	9.36
7H	601,597,413	188	593	94.63	0.50	14.84
Total	3,902,827,061	1129	3662	645.56	0.57	16.02

^a^Chromosome number

^b^Total physical length of the chromosomes (bp)

^c^Number of bin markers

^d^Single Nucleotide Polymorphism

^e^Total genetic distance of chromosomes (cM)

^f^Average genetic distance between markers (cM)

^g^Maximum gap between markers (cM)

**Table 4:** Linkage map gap statistics

Chr^a^	< 5 cM	5to10 cM	10to20 cM	> 20 cM	ratio
1H	103	0	0	0	100.00
2H	297	2	1	0	99.00
3H	176	3	2	0	97.24
4H	113	2	1	0	97.41
5H	123	2	1	0	97.62
6H	106	4	0	0	96.36
7H	184	2	1	0	98.40
Total	1102	15	7	0	98.00

^a^Chromosome number

### Map quality validation

**Fig. 4:**
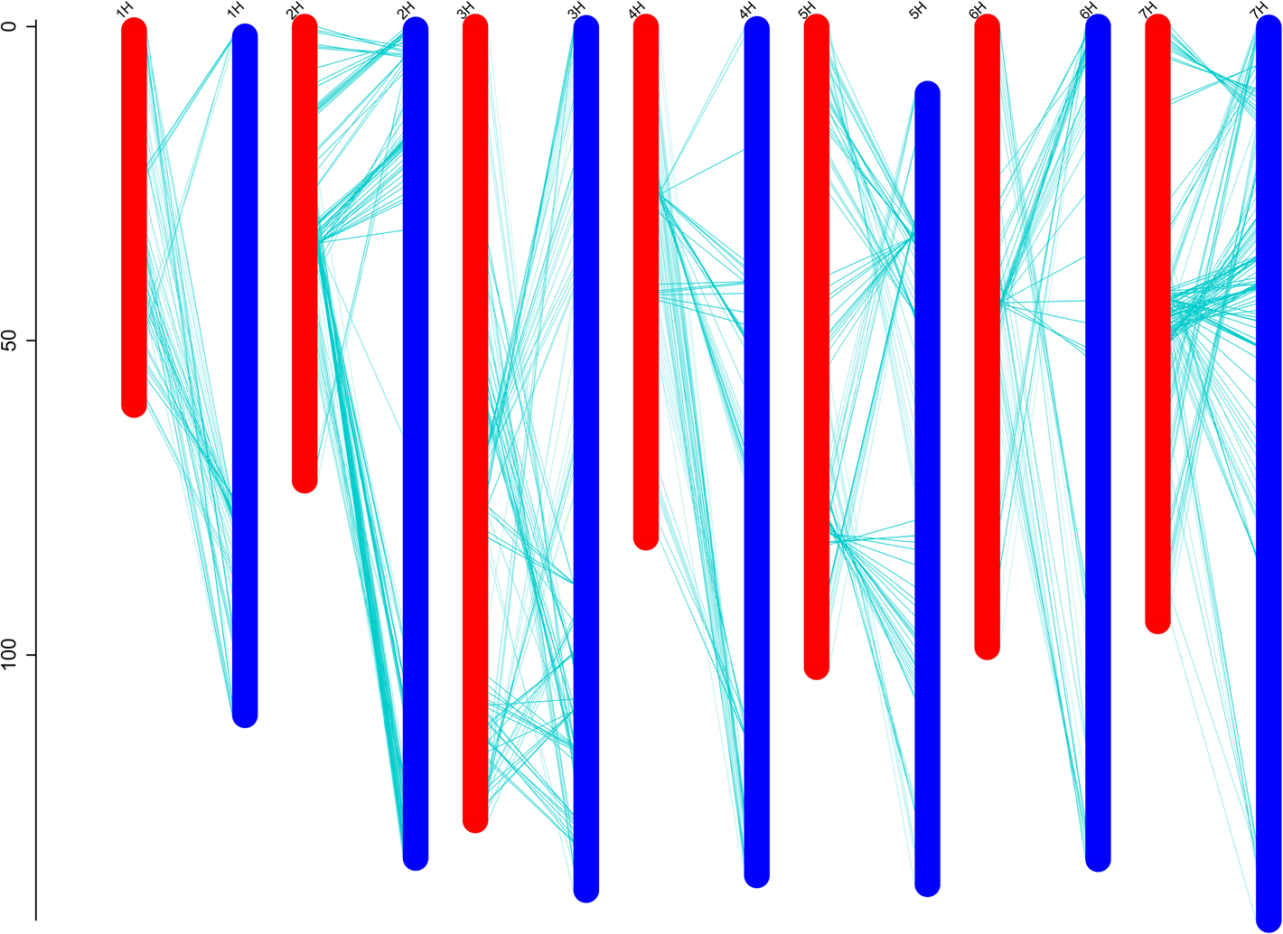
The physical map and genetic linkage map. Note, the x-axis is the chromosome number and the y-axis is the genetic distance (in cM). The genetic map is shown in red, the physical map is in blue, and the green line shows the position of each marker on the genetic map and the physical map

A co-linearity analysis indicated that most of the markers in the linkage groups were consistent with the hulless barley reference genome, indicating that our estimation of genetic recombination rate was highly accurate (Fig. 4). The genetic map that was constructed with the SNP markers discovered in the GBS-Seq analysis had sufficient coverage throughout the barley genome. Most of the SNP loci on the linkage map were in the same order as those on the corresponding chromosomes of the physical map of the hulless barley genome.

**Fig. 5:**
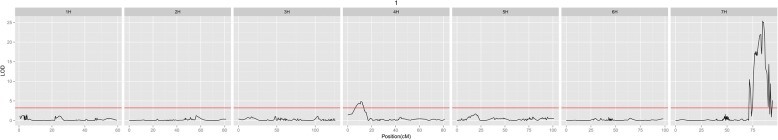
Linkage analysis of *Psc* in hulless barley among all the linkage groups. Note: the x-axis is the genetic distance of each linkage group, the y-axis is the physical position of bin markers against LOD scores and the red line is the threshold LOD value as determined by a permutation test; 1~ 7, seven linkage groups

### Phenotypic features of DH lines and linkage analysis of *Psc*

A DH population of 298 offspring from Nierumuzha × Kunlun10 was used in this study. The seed coat color of offspring ranged from deep purple to yellow gradually (Additional file 1). We divided them artificially into four categories according to the result of Wanseen seed test system SC-G (27 deep purple seeds, 68 purple seeds, 133 light purple seeds, and 70 yellow seeds) (Fig. 1b) [28]. Linkage analysis was based on the phenotypic data for *Psc* in DH lines at a LOD of 3.2. A total of 5 loci for *Psc* were identified: one locus on 4H, explaining 3.79% and four loci on 7H (Fig. 5), explaining 13.64%, 17.56%, 23.86%, and 15.26% of phenotypic variation, respectively. The locus peak positions were located on 10.91 cM (4H), 71.81 cM (7H), 77.41 cM (7H), 84.61 cM (7H), and 90.61 cM (7H), respectively, and were located within the marker intervals of 4H-bin857 to 4H- bin863 (275 genes), 7H- bin1533 to 7H- bin1532 (57 genes), 7H- bin1383 to 7H- bin1384 (60 genes), 7H- bin1377 to 7H- bin1372 (248 genes), and 7H- bin1370 to 7H- bin1369 (189 genes) (Table 5).

**Table 5:** Linkage analysis of *Psc*

Site name	Chr^a^	LOD peak	Position (cM)	99% CI (cM)^b^	R^2^(%)^c^	Additive effect	Left marker^d^	Right marker^e^	Gene number
*Psc-1*	4H	4.81	10.91	10.5–13.8	3.79	0.2779	bin857	bin863	275
*Psc-2*	7H	10.45	71.81	71–73.1	13.64	−0.376	bin1533	bin1532	57
*Psc-3*	7H	17.57	77.41	77.3–78.5	17.56	0.443	bin1383	bin1384	60
*Psc-4*	7H	25.37	84.61	84.5–86	23.86	0.5598	bin1377	bin1372	248
*Psc-5*	7H	14.34	90.61	90.5–90.7	15.26	−0.3707	bin1370	bin1369	189

^a^Chromosome number

^b^99% confidence interval for site length

^c^Proportion of phenotypic variation explained by each site

^d^Left boundary markers for each site

^e^Right boundary markers for each site

### Prediction of candidate genes

Five structural candidate genes and one regulatory factor related to flavonoid or anthocyanin biosynthesis were identified in the *Psc* regions within the loci (Additional file 2) according to the barley gene annotation database accessible at TrEMBL, NCBI, KEGG and Swiss-Prot. The candidate gene MLOC_6177, located on chromosome 7H (71.00–73.10 cM, see Additional file 3), was annotated to an uncharacterized protein in barley in TrEMBL and flavonoid 3′-monooxygenase (*F3*’*M*) in Aegilops (GenBank Accession Number XM_020332250.1) in KEGG and Swiss-Prot. MLOC_71630 located on chromosome 7H (77.30–78.50 cM, see Additional file 4), was annotated to an uncharacterized protein in barley at TrEMBL and 2-hydroxyisoflavanone dehydratase (*HID*) in Aegilops in Swissprot. MLOC_62096 located on chromosome 7H (84.50–86.00 cM, see Additional file 5), was annotated to anthocyanidin 3-O-glucoside 2′′-O-xylosyltransferase (*UF3GT*) in barley in Swiss-Prot. MLOC_38343 located on chromosome 7H (84.50–86.00 cM, see Additional file 5), was annotated anthocyanidin/flavonoid 3-O-glucosyltransferase (*UFGT*) in barley in Swiss-Prot. MLOC_32012 located on chromosome 7H (90.50–90.70 cM, see Additional file 6), was annotated to an uncharacterized protein in barley in TrEMBL and malonyl-coenzyme: anthocyanin 5-O-glucoside-6′′′-O-malonyltransferase (*5MAT*) in Aegilops in Swiss-Prot. MLOC_6171 located on chromosome 7H (84.50–86.00 cM, see Additional file 5), was annotated to an uncharacterized protein in barley in TrEMBL, but anthocyanin regulatory C1 protein (*Ant1*) in barley in NCBI.

## Discussion

Linkage analysis is an efficient way to analyze some important traits in barley molecular breeding [29, 30]. The quality of genetic maps, however, can significantly affect the accuracy of gene mapping. This increases the density of markers distributed around the entire genome, which can improve the resolution of genetic maps [31–33]. SSR, one of the traditional gene mapping methods, was used in hulless barley, but the number of SSR markers is limited. Meanwhile, no markers were sufficiently close to key traits to ensure reliable detection after exhaustively testing [34]. GBS is a fast, efficient, informative, and cost-effective strategy for SNP discovery, genetic linkage map construction, and genotyping [35]. In this study we used a combination of three restriction enzymes (*HaeIII, MseI*, and *EcoRI*) for GBS library construction. *MseI* recognizes a 4-bp restriction site (TTAA). It also has a higher distribution frequency in the Chinese jujube (*Ziziphusjujuba*) genome. The use of two additional enzymes, namely, *EcoRI* and *HaeIII*, was performed to further digest the fragments after *MseI*. The three restriction enzyme combination improved the efficiency of GBS by increasing sequencing depth, number of tags, and genome coverage. This combination also allowed the detection of suitable regions for targeted fragments [36]. The present study generated the first high-density genetic map of hulless barely using the GBS technology. A total of 490.07 Gb raw sequencing data and 96.93% of clean data were mapped to unique positions on the reference genome. We were able to cost-effectively genotype 3662 SNPs with 1129 bin markers with an average distance of 0.57 cM in 300 samples (2 parents and 298 offspring) of hulless barely using the GBS technology. Such a high-density linkage map will likely be a valuable resource for genomic analyses and fine-scale gene mapping in hulless barely.

The inheritance of the purple pericarp and lemma has been studied with inconsistent results up to date. Woodward and Thieret [37] obtained 3:1 ratios of purple and nonpurple seeds from 28 crosses of “purple” × “nonpurple” barley genotypes, and 9:7 ratios from the crosses in which both parents were non-purple seeds, indicating a two-factor inheritance. To simplify the nomenclature, previously reported symbols were dropped and *Pre1* and *Pre2* symbols were used for C, c, and P, p, located on 1H and 2H, respectively [38]. In this study we found 298 offspring from Nierumuzha × Kunlun10 seed coat colors range from deep purple to yellow gradually. In order to shorten the distinguish time of breeding in future, we divided them artificially into four categories: deep purple, purple, light purple and yellow. We thus mapped *Psc* as a qualitative trait using the GBS linkage map. The major *Psc* loci were located on chromosome 4H (1 locus) and 7H (4 loci). Previous studies have indicated that seed coat color of barley was located on chromosomes 2H [38] and 4H [13] at a genetic distance greater than 10 cM. In this study, five loci were detected on chromosomes 4H and 7H that one explained 3.79% (length 3.3 cM) of the phenotypic variation, whereas the other four (71.00 cM–90.70 cM) accounted for 13.64% (length 2.1 cM), 17.56% (length 1.2 cM), 23.86% (length 1.5 cM), and15.26% (length 0.2 cM) of the phenotypic variation according to the high-resolution map (Table 5). There are 23 bin markers between 70.944 cM and 92.072 cM (bin1533-bin1369) on 7H, and the average marker density is 0.92 cM. The average gap is 0.746 cM except the largest one (5.455 cM) between qPSC-2 and qPSC-3 (bin1532-bin1383) (Additional file 7). In order to avoid the wrong judgment in this region, we re-tested candidate genes according to gene annotations by Swissprot, TrEMBL, and the Kyoto Encyclopedia of Genes and Genomes (KEGG) analysis, but didn’t found new ones.

Seed coat color is thought to be associated with the synthesis of anthocyanins. Anthocyanin biosynthesis is well characterized at the enzymatic, genetic, and production levels. These structural genes can be separated into two categories: those of the late flavonoid biosynthetic pathway, including anthyocyanidin synthase (*ANS*), dihydroflavonol 4-reductase (*DFR*), UDP-sugar: flavonoid-3-O-glucosyltransferase (*UFGT*), and flavonoid-5-O-glucosyltransferase (*UF5GT*) [39], and those of the early flavonoid biosynthetic pathway, including chalcone synthase (*CHS*), chalcone isomerase (*CHI*), phenylalanine ammonia-lyase (*PAL*), flavonoid 3-hydroxylase (*F3H*), and flavonoid 3′-hydroxylase (*F3*′*H*). Shoeva et al. identified the *Ant2* gene and showed that the mRNA levels of flavonoid biosynthesis structural genes *CHS*, *CHI*, *F3H*, *DFR*, *F3*′*H*, *ANS*, and the regulatory gene *ANT2* were higher in purple barley than in yellow barley, as indicated by qRT-PCR [3]. Even though the genes involved in anthocyanin biosynthesis have been identified, their relation to *Psc* in hulless barley is currently unknown. In this study, some structural candidate genes and regulatory factors related to flavonoid or anthocyanin biosynthesis were identified in the *Psc* regions by gene annotation. Sequence analysis showed the nucleotide sequence of MLOC_6177 from hulless barley to be 99% homologous with a predicted protein gene NIASHv2048J10 (GenBank Accession Number AK366933.1) of barley and 95% homologous with *F3*′*M* of Arabidopsis (GenBank Accession Number AK366933.1), respectively. *F3*′*M* belongs to the cytochrome P450 family and is related to flavonoid biosynthesis [40]. The nucleotide sequence of MLOC_71630 from hulless barley was 99% homologous with a predicted protein gene NIASHv1124H04 of barley (GenBank Accession Number AK360742.1) and 92% homologous with 2-hydroxyisoflavanone dehydratase gene of Aegilops (GenBank Accession Number XM_020315035.1). The co-action of a *HID* and 2-hydroxyisoflavanone synthase (*IFS*) produces flavone from flavanone, which is related to isoflavone biosynthesis [41]. The nucleotide sequence of MLOC_62096 from hulless barley was 99% homologous with *UF3GT* of barley (GenBank Accession Number AK358154.1). UF3GT contributes to the last few steps in anthocyanin biosynthesis by converting cyanidin 3-O-xylosyl (1- > 2) glucoside. It can use 3-O-glucosylated anthocyanidins and uridine diphosphate (UDP)-xylose as substrates [42]. The nucleotide sequence of MLOC_38343 from hulless barley was 99% homologous with *UFGT* of barley (GenBank Accession Number X15694.1). *UFGT* is involved in the anthocyanin biosynthesis pathway, which forms part of pigment biosynthesis [42]. The nucleotide sequence of MLOC_32012 from hulless barley was 92% homologous with *5MAT* of Aegilops (GenBank Accession Number XM_020296576.1). *5MAT* is involved in later reactions in anthocyanin modification [43]. The nucleotide sequence of MLOC_6171 from hulless barley was 99% homologous with *Ant1* of barley (GenBank Accession Number KP265979.1). *Ant1* can act as a trans-acting factor (MYB related family protein) that controls the expression of genes involved in anthocyanin biosynthesis and regulates the expression of at least three structural genes: dihydroflavonol reductase, chalcone synthase, and flavonol O_3_ glucosyltransferase [44]. Regulatory factors such as MYB, bHLH and WD40 proteins [45] primarily control anthocyanin biosynthesis, as well as a series of structural genes (enzymes). In addition to *Ant1*, we also discovered some other transcription factors including bHLH69, bHLH82, bHLH96, MYB39, WDR, TRAB1 and IND, etc. The relationship between transcription factors and anthocyanin biosynthesis still requires further validation. Also, the function of the above-mentioned annotation genes need further study.

## Conclusion

A high-density genetic linkage map was constructed using the GBS method. The linkage map contained seven linkage groups with a low inter-marker distance. This high-density linkage map can serve as a foundation for obtaining additional genetic knowledge of hulless barley. Five loci for *Psc* were identified and will be useful in marker-assisted selection studies for this important agronomic trait. Using linkage analysis and gene annotation, five structural candidate genes and one regulatory factor related to flavonoid and anthocyanin biosynthesis were identified. These genomic resources may also play an important role in genetic breeding studies and future whole-genome sequencing projects in hulless barley.

## Additional files

Additional file 1: The phenotype pictures of female, male and four types of seed coat color. (JPG 267 kb)

Additional file 2: All candidate genes sequences. (DOCX 18 kb)

Additional file 3: Gene annotations of 7H within the region encompassing 71.00–73.10 cM. (XLSX 15 kb)

Additional file 4: Gene annotations of 7H within the region encompassing 77.30–78.50 cM. (XLSX 15 kb)

Additional file 5: Gene annotations of 7H within the region encompassing of 84.50–86.00 cM. (XLSX 30 kb)

Additional file 6: Gene annotations of 7H within the region encompassing 90.50–90.70 cM. (XLSX 25 kb)

Additional file 7: The marker information between bin1533 to bin1531of 7H. (DOCX 16 kb)

## Abbreviations

*5MAT*: Malonyl-coenzyme: -O-glucoside-6″‘-O- malonyltransferase
*ANS*: Anthyocyanidin synthase
BWA: Burrows-wheeler aligner
CHI: Chalcone isomerase
CHS: Chalcone synthase
CP: Cross pollination
*DFR*: Dihydroflavonol 4-reductase
DH: Doubled haploid lines
*F3*′*H*: Flavonoid 3′-hydroxylase
*F3*′*M*: Flavonoid 3′-monooxygenase
*F3H*: Flavanone 3-hydroxylase
GBS: Genotyping by sequencing
*IFS*: 2-hydroxyisoflavanone synthase
LOD: Logarithm of odds
*PAL*: Phenylalanine ammonia-lyase
PE: Paired-end
*Psc*: Purple seed coat color
QC: Quality control
SNP: Single nucleotide polymorphism
*UF5GT*: Flavonoid-5-O-glucosyltransferase
*UFGT*: Anthocyanidin/ flavonoid 3-O-glucosyltransferase
*UFGT*: UDP-sugar: flavonoid-3-O-glucosyltransferase
