# Supplementary figures and images for: Construction of a high-density genetic map: genotyping by sequencing (GBS) to map purple seed coat color (Psc) in hulless barley
Source: Hereditas. 2018 Nov 17;155:37. doi: 10.1186/s41065-018-0072-6 (PMC6240233; doi:10.1186/s41065-018-0072-6)

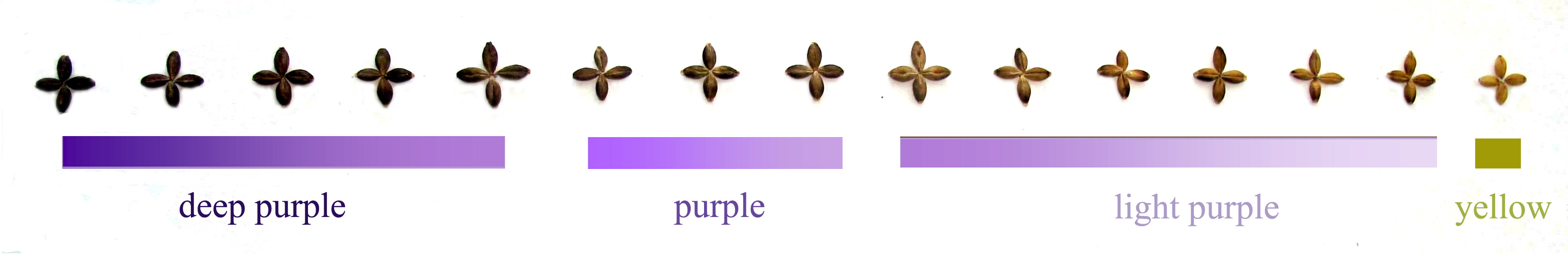

Supplement: Supplementary file 1 — The phenotype pictures of female, male and four types of seed coat color. (JPG 267 kb) [file 41065_2018_72_MOESM1_ESM.jpg]
