# Supplementary material for: Construction of a high-density genetic map: genotyping by sequencing (GBS) to map purple seed coat color (Psc) in hulless barley
Source: Hereditas. 2018 Nov 17;155:37. doi: 10.1186/s41065-018-0072-6 (PMC6240233; doi:10.1186/s41065-018-0072-6)
Supplement: Supplementary file 2 — All candidate genes sequences. (DOCX 18 kb) [file 41065_2018_72_MOESM2_ESM.docx]

**Supplemental Data1** Sequences of all candidate genes.

>MLOC_6177

TTCACCTGCACAGTTTCCATTCCTATCATCGCTCCAGTGAGTAGTAGGCGACACTAAACCATCCGAAACGCAAATGGTGGTAGGCATACCGGTAGGTAGGCGAAAGCTGCAAGAGACCCATATTGTAATAGTTGGTATGCAAAAGCTGTGGCTGAGCTTTGCCGGTTGAGACGTGTCAGCTGTGCAGTTTTGCCGGTCACGTCCACCTACCGCCTTCACCTTGAACCCCATGCATGCATACATGAACACTCGAGCGCCTGCACTGCATGCATATCGAACAGAACAATGCATCTCGTCGCCATGGATGTCCCTCTCCCGTTGCTCCTCTCCACCTTGGCCGTTGCCGTGGGCATATGCTATGCGCTCTTCTCCTTCCGCGCCGACAAGGGGCGCGCGCCGCTGCCGCCGGGGCCGAGGGGCTGGCCGGTGCTGGGGAACCTCCCGCAGCTCGGCGGCAAGACGCACCAGACATTGCACGAGATGTCCAAGGTGTACGGTCCCGTGCTCCGGCTCCGGTTCGGCAGCTCCGTCGTGGTGGTGGCCGGGTCGGCCGCCGCGGCCGAGCAGTTCCTCCGCATACATGACGCCAAGTTCAGCAGCCGGCCGCCCAACTCCGGCGGCGAGCACATGGCGTACAACTACCAGGACGTGGTGTTCGCGCCCTACGGCCCCCGGTGGCGCGCGATGAGGAAGGTGTGCGCCGTGAACCTCTTCTCGGCCCGCGCGCTCGACGACCTCCGCGGGTTCCGCGAGCGGGAGGCCGCGCTCATGGTGAGGTCCCTTGTTGATGCTGCCTCCGGCGGCGGGGTGGTGGCCGTCGGCAAGGCGGCGAACGTGTGCACGACCAACGCCCTCTCGCGGGCGGCGGTGGGACTGCGGGTGTTCGCCGCGGCCGGCACCGAGCTTGGCGCCCAGGAGTTCAAAGAGATCGTGCTGGAGGTGATGGAGGTTGGGGGCGTGCTTAACGTCGGGGACTTCGTGCCGGCGCTGCGGTGGCTCGACCCGCAGGGGGTCGTTGCCAGGCTGAAGAAACTGCACCGCCGGTTCGACGACATGATGAACGGGATCATCGCCGAGAGAAGGGCCGGAGGTAGCACGGCCGGCGACGAGAAGGAAGGTAAGGACCTGCTCGGCCTGCTTCTCGCAATGGTGCAGGAAGACAAGTCCCTCACCGGCGGCGAGGAGGACAAGATCACCGACACAGACGTCAAGGCGCTCATACTGGTAACAATCTAGCACTTCGTTGCCGTGGCTGCGACGTGTGATCCACAACCGCAGTCATATAATGTGCTTGATTAATTAACCCTCCCTGCATGCATGCATGCATGCACGCACGCAGAACTTGTTCGTGGCCGGCACGGAGACGACGTCGACGATAGTGGAGTGGGCAGTGGCGGAGCTGATCCGGCACCCGGACATGCTGAAGCGGGCGCAGGAGGAGCTGGACGCCGTCGTGGGCCGGGACAAGCTCGTCGCGGAGTCGGACCTGCCGCGCCTCGCCTTCCTCGGCGCGGTCATCAAGGAGACGTTCCGGCTGCACCCGTCGACGCCGCTGTCGCTGCCGCGGATGGCCTCCGAGGAGTGCGAGGTCGCCGGCTACCGCATCCCCAAGGGCACGGAGCTGCTGGTCAACGTGTGGGGCATCGCCCGCGACCCGGCCCTGTGGCCCGACCCGCTCGAGTTCCGGCCCGCCAGGTTCCTCCCGGGAGGGACGCACGCCGACGTCGACGTCAAGGGCGGCGACTTCGGGCTGATCCCGTTCGGGGCCGGCCGGAGGATCTGCGCGGGCCTCAGCTGGGGCCTGCGGGTGGTCACCGTGACGACGGCCACGCTGGTGCACTCGTTCGACTGGGAGCTGCCGGCGGGCCAGACGCCCGGCAAGCTCAACATGGAGGAGGCCTTCTCTCTGCTGCTGCAGCGGGCCGTGCCGCTCATGGTCCACCCCGTGCCCAGGCTGCTCCCGTCGGCATACCAAATTGCGTAGAAACGAACAAGACAGAGGACAAAGTATGCTGCTTTTTTTTTTCTTTTTTCTGAGGGGACAAACTATATATGCTGCTGATTATCGTGATGAGCTATGGAGGACAAACTCCAGTTGTGCCATGTAACTGTTGTAGATGGGCGTGCCTCACCGTTTTCGACTAAAATAATTCCACCTACTTGTGTATTGCTTTGCACTCATTAGTAACAATGGAAAATAACATTTTATGTACCAAGAAATATGGGAGCG

>MLOC_71630 GAAAAAGACCCCTCCCGATGCCCACAATGATCGGCACCGACCGCGCGGATGGTATGGTGGTCATCTCCACTCTCGCGCACACCACTCTGCTCTGCCGCGCACCAAACCATCAAAAAGTGCTGCATCGCCATTGTTGCCACCACAGACCACACCAACCGTTCGGTCCATCAACATCCGGCATCCTCCACTCCTCCACTTACTCCATTATTACATAGTGCCGGCGATCGAGACCCGAGAGCAAAGCCCCGGAGCGACGGCACGAGCGATCTAGCCCACGAACGACCAAACACGCGACCACAAGGGCTCAGCCAGCTCGTACCAATGGCGGCGACGGGCGGCGCGGACTCGGAGGTGCACTTCGACTTCTTCCCGCTCGTCCGCCAGTACAAGAGCGGCCGCGTGGAGCGGTTCATGAACTTCCCCCCGATCCCGGCCGGCGTCGACCCCGCCACGGGCGTCGCCTCCAAGGACGTCGTCATCGACCCGGCCAACGGCCTCTGGGCGCGCGTCTTCCTCCCGCCCGGCGGCCACGACGGCAGCAAGCTCCCCGTCCTCGTCTACTTCCACGGAGGCGCCTACGTCATCGGCTCGGCGTCCGACCCCATGACGCACAACTACCTCAACGGCCTAGTCGCCGCAGCGAACGTCGTCGCGGTGGCGCTCGAGTACCGCCTCGCGCCGGAGCACCCGCTCCCGGCCGCCTACGACGACTCCTGGGAGGGGCTCAAGTGGGTGGCGTCCCACGCCACGGCCGCCGCGGCCGACGGCGCGGAGCCGTGGCTGGCCGACCGCGGCGACTTCTCCCGCGTGTTCCTGGCGGGCGGCAGCGCCGGGGGCACCATCGCGCACGTCATGGCCGTGCGCGCCGGCGAGCAGCAGGGCGCCCTCCCCGGGTTCGGCATCAGGGGGACCATCGTGGTGCACCCTTACTTCAGCGGCGCGGCGGCGATCGGCAAGGAGGCGACCACGGGGAAGGCGGAGAAGGCCAAGGCCGACGCCTTCTGGCGGTTCCTCTACCCGGGCTCGCCGGGGCTGGACGACCCGCTGTCCAACCCGTTCTCCGAGGCGGCCGGCGGCAGCGCGGCGCGCATCGCCGGCGACCGCGTGCTCGTCTGCGTCGCCGAGAAGGACGGCCTCCGGGACAGGGGCGTCTGGTACTACGAGAGCCTCAAGGCGAGCGGCTACGCCGGCGAGGTGGAGCTGCTCGAGTCCGTCGGCGAGGACCACGTCTTCTACTGCATGAAGCCGCGGTCCGAGAGAGCGATCGAGCTGCAGGACCGCATCCTCGGCTTCCTACGCAAGTGAAGGGAAGTTCCCGTGATGGAACAATTTTATCGCCATTGTTGTTCCTCTGTTTGTTTGAGCTCGAGATATCGATCTCTCCCTTTAATTTGTTGTCCGTCGATTCTCGACTTCTGGTCTCGCCGATGCAGTCGACTGATGTTTATGTGCTCGATTCCATGGCGAGGAGAGCTAGTCGGTTTCTTAGACCATCTGTGATGAACTTGTTGTTTTCAACCATCTTTCCTGGTAGAGGCCGAAAGCAATGAATAAAGCTCTTTCATTTCTTTTAGTGCTAATCATCGTGTGAAAAGTGATGGATCACGTTTGATGTTTTGAAGAGAAGACGATATTCTTAGCCTTGTAATTGTGTTTTAAACTGCTAAAAAAAGGAAAATGCT

>MLOC_62096 CACTGCCCACACAGCAACACTTACGCCAACACCAAGCACCAAGCCTCTTCCTGTTTTATTTTGCTCCACCGATCCGTTGCGAGCAGATGGAAATGGAAGCAGCGGATGCCGGCGCCGGCGAGCTGGAGGTGGTTGTGTTCCCGTGGCTGGCATTCGGGCACATGATCCCGTTCCTGGAGCTCTCCAAGCACCTCGCGGCCAGAGGCCACGCCGTGGCCTTCGTGTCCACGCCCCGGAATCTCGCCAGGCTCCCGCCCGTTCCGGCCGGTCTTTCCACCCGCCTCCGGTTCGTGCCGCTGCCGCTGCCCGCCGTGGAGGGGCTGCCGGAGGGCGCCGAGGCCACGTCTGACCTGCCGCCAGACAAGGTCGGGCTCCTCAAGAAGGCCATGGACGGCCTCGCCGACCCACTCGCGGCATTCCTCGCCGCCGGAAGGAGGCCCGACTGGATCCTCCACGACTTCTGCCACCACTGGGTCCCTCCCATCGCCGACCAGCACAAGGTGGCGTCCGCCACGTTCCTCATCTTCCAGGCCGCCTTCTTGGTCTTCGTGGGGCCACGGTGGGCAAACACCGCACACCCGCGCACGGAGCCGGAGCACTTCGCCGAGGCACCCAGGTGGATTCCCTTCCCGTCCACCACCTTCTTCCGCCGCCACGAGACTCAGTGGATCACCGACGCCTTCCGTACCAATGCATCTGGCGTGTCCGACATGGACCGCTGGTGGCAGGTCTTGGAGCACAGCCGCCTCACCATCCACCGGAGCTGTGAGGAATTAGAACCCCGGATGTTCGGCCTCCTATCCGATCTCTTCCGGAAGCCCGCTGTGCCCGCCGGGATCCTGCTGCCGGGGGCGTCCGACGGCCTTGACGAAGACCACTGGCAGAGCACCTCAGGCGGCGTCGCCCGTCCGCAGGTTCTGCGATGGCTCGACGACCAGCCTCCCAAGTCTGTCATCTACATTGCGCTGGGAAGCGAGGCGCCGCTGACGCCAGAGAACGCCCATGAACTTGCGCTCGGTCTGGAGCTCGCCGGTGTGCGCTTCCTCTGGGCACTGCGCAAGCCGGCAGGCACCGGCAGCGACGATGAGCTTCTGTTGCCGGCCGGGTTCGAAGAGCGGACGCGGGACCGCGGGGTGGTTTGCACGGGGTGGGTGCCGCAGGTGGAGGCACTGGCGCACTGCGCCACGGGCGCGTTCTTGTCGCACTGTGGCTGGGGCTCCACCATCGAGAGCCTCTCCATCGGGATCCCGCTGGTCATGCTCCCGTTCGTCGTCGACCAGCCCTTGATCGCGCGGGCGATGGCTGAGAGAGGGATCGGCGTGGAGGTGGCGAGAGATGAGAACGACGGTTCGTTTGACAGGGACGGCGTTGCGGTGGCGGTGCGGCGCGTCATGGTGGAGGAGCAGGGAAAGGTGTTCGCGACCAACGTGAAGAAGCTGCAAGAGATTCTAGTGGACCAGAGACGCCAAGAGCACTACATCGACGAGCTCGAGGAGCACCTGAGACGCTACAGAGACGTCTAATATACTCCGTAATGAGTAATGCTACACGTACAAAGGCTTACGTAAAGATTTTATGTACAAACTGATGTGTAAGATTATGATTAGTAATTGAGGTATGAGGGGG

>MLOC_38343

TCGCGGCCGGGCTGGAGGCGAGCGGCGCGCCGTTCCTGTGGTCGCTGCGCGAGGAGTCGTGGCCGCTGCTCCCGCCGGGGTTCCTGGAGCGCGCGCCGGGCCTCGTGGTGCCGTGGGCGCCGCAGGTGGGCGTGCTGCGGCACGCCGCGGTCGGCGCGTTCGTGACGCACGCCGGGTGGGCGTCGGTGATGGAGGGAGTGTCCAGCGGCGTGCCCATGGCGTGCCGGCCCTTCTTCGGCGACCAGACGATGAACGCGCGGTCGGTGGCCAGCGTGTGGGGCTTCGGCACGGCGTTCGACGGGCCGATGACGCGCGGCGCCGTGGCAAACGCGGTGGCGACGCTGCTGCGCGGGGAGGATGGGGAGCGGATGAGGGCAAAGGCGCAGGAGCTGCAGGCCATGGTGGGCAAGGCGTTCGAGCCCGACGGCGGCTGCAGGAAGAACTTCGACGAGTTTGTCGAGATAGTTTGTCGGGTGTGATCGTGTACTCGTACAAATGCTTAGAACTCTATGGGACCGATTACGTGTAAGTCGACCGTCTTTTTTATGTGTGTGTAAGTCACTTTGTTTTTACCGTCAGATATCCATCCGACGGCGTGATCTTCTTCCTCCCAACCGAGTCCTGACCGCAGCCATCCACCAGCTGTGACCGCCCCGTACTCCCCTCGACCCTCCCCACCACGGTTCTGGTCGTCGCCCTGGTCCATCCGTCTAAGGGCCAGTTTTTTTTTGCCAGACTTTTTATAAGCTGCCTTCTA

>MLOC_32012

CGACCGCGATGGCGCCCGGGCCACTTAACCTTGTGACGGTCATGGAGCAGTTCCATGTCTCGCCGTCGCCGTCACCACCGGCCGAGATGCCACGGGCGCTGCCGCTCACCTTCTTCGACCTCGCATTCTGGGACGTCCCGCCCGTGCAGCGCCTCTTCTTCTACGACAACGCCGACCTCCTCGGCGCCCCCGAGTTCCTCCTCCACGAGCTGCCTCTGTTCAAGAAGTCCCTGGCCGCCGCGCTGCACCACTTCTACCCCTTGGCCGGGACGCTGGTCCGCAGCATACCGGAGGCCGGGGCGCCCGAGGTCGTTTTCTCAGAAGGCGACTCTGTCCGCCTGACTGTTGCGGTCGGCGGCGACGACTTCCAGGACCTCGCCGGCGACCAAGCGCGCGACACCGCGAGGCTCCGTCCGCTGCTGCCTTCCCTGCCGGAACATGGCGATGGCGGTGGTTCTCGGTGCAGCACTCAGGACGTCTTTGCCGTCCAGGTCACCCTGTTCCCTCACGCCGGTTTGTGCATTGGCACAACGCTACACCACGCCGTGGCCGACGGTTCCAGCTACGTGCACTTCATGAGGACGTGGGCAGCCATCCACCGCCTCGGCCCCGAGCGCGGCGGGAAGTGGGCCGCGCCCCCGCTGCTCGACCGTAGCGTCGTGCGAGACGACAACGGGCTCCGCGAGGTGTTTCTCCGCGACCACCGGGCTCTCGCGGCGGCCGGCGGCAAGCGGCCCCACGACTGGGACCTCAGCAAACGCCCGGGCGCCGCTCACCTCGCGACGTTCCGG

>MLOC_6171

CGAAATCAAGAACTACTGGAACAGCACGCTCGGCAGGAAGGCGCTCCCCGCCCGGCCCGCCATTGCCGCGGCGAGGACCATCGCCTCCGGATCCTCCAGCTCCACGGGGAGCGCCGCGGCGGCGCTCTCCACCTCAGTCCTTGCCGTCCTCCACGCCGCGGCGCCTTCGTCGTCGCCGGCCGCTGTGTGGGCGCCCAAGCCCGTGAGGTGCACTGGCGGCCTCTTCTTCCGCCGGGAGACGCCGCCGCCCGCGCCGGTCGTCGAGGAGACGCGGACCAGGGGAGAAGAGCCACCATTTCATGCCTGCAGCGGTAGCAGCTCGGCCTCGGAGGCGTCTCCGGCCGAGCTTTGCTCGTCGGGGTCCGGCGGGGGAGACTGGATGGACGACGTGAGAGCCCTGGCGTCGTTCCTCGAGTCCGACGAGGAATGGCTCAAGTCCCTGCACATGGCCGGTTAAACCTCTCGATCACACGTGATCGATCGAGCTCGCTCACATGTTAAGTACGTGGTGCGCCGTTGTACGTACGCGCTTCCTCAAAGGGAAACTGTGCGTACACGGTATACGTACTACGTGGCTGGCTAGCTCCCGTACGTTGCGAGTGTACTGAGTTGTTCGTGTAGACGTTAGTTTTGAGGGAAAAAAATGATAAGGTGGCGTCGTTCCTCACATGTCCACGACAGCGCAACAGAAAAGGTCTAGGACAGTACTACGCGTAGGCGCGCCGGCGCGAGTGCTAGCACCGGTTGCAGCGTGACCGTCAGATCTGCAGGGGGCTGTCAGATATCTTCATGCAACAAATTTTGATGCTATG
