# Supplementary material for: Construction of a high-density genetic map: genotyping by sequencing (GBS) to map purple seed coat color (Psc) in hulless barley
Source: Hereditas. 2018 Nov 17;155:37. doi: 10.1186/s41065-018-0072-6 (PMC6240233; doi:10.1186/s41065-018-0072-6)
Supplement: Supplementary file 7 — The marker information between bin1533 to bin1531of 7H. (DOCX 16 kb) [file 41065_2018_72_MOESM7_ESM.docx]

Additional file 7 The marker information between bin1533 to bin1531of 7H

| Chr^a^ | Bin Markers | Genetic Distance/cM | Gap/cM | QTL name |
| --- | --- | --- | --- | --- |
| 7H | bin1533 | 70.944 | 0.711 | qPSC-2 |
| 7H | bin1532 | 71.841 | 0.897 | qPSC-2 |
| 7H | bin1383 | 77.296 | 5.449 | qPSC-3 |
| 7H | bin1382 | 77.422 | 0.126 | qPSC-3 |
| 7H | bin1387 | 78.439 | 1.017 | qPSC-3 |
| 7H | bin1384 | 78.544 | 0.105 | qPSC-3 |
| 7H | bin1388 | 78.818 | 0.274 |  |
| 7H | bin1386 | 79.278 | 0.46 |  |
| 7H | bin1385 | 79.32 | 0.042 |  |
| 7H | bin1381 | 81.859 | 2.539 |  |
| 7H | bin1376 | 83.144 | 1.285 |  |
| 7H | bin1375 | 83.271 | 0.127 |  |
| 7H | bin1374 | 83.494 | 0.223 |  |
| 7H | bin1373 | 83.747 | 0.253 |  |
| 7H | bin1377 | 84.315 | 0.568 | qPSC-4 |
| 7H | bin1379 | 84.585 | 0.27 | qPSC-4 |
| 7H | bin1380 | 84.776 | 0.191 | qPSC-4 |
| 7H | bin1378 | 85.54 | 0.764 | qPSC-4 |
| 7H | bin1372 | 87.201 | 1.661 | qPSC-4 |
| 7H | bin1371 | 87.693 | 0.492 |  |
| 7H | bin1370 | 88.917 | 1.224 | qPSC-5 |
| 7H | bin1531 | 90.569 | 1.652 | qPSC-5 |

^a^ Chromosome number
